# System analysis based on the migration- and invasion-related gene sets identifies the infiltration-related genes of glioma

**DOI:** 10.3389/fonc.2023.1075716

**Published:** 2023-04-06

**Authors:** Shuang Shi, Jiacheng Zhong, Wen Peng, Haoyang Yin, Dong Zhong, Hongjuan Cui, Xiaochuan Sun

**Affiliations:** ^1^ Department of Neurosurgery, The First Affiliated Hospital of Chongqing Medical University, Chongqing, China; ^2^ State Key Laboratory of Silkworm Genome Biology, Southwest University, Chongqing, China; ^3^ Cancer Center, Medical Research Institute, Southwest University, Chongqing, China

**Keywords:** glioma, infiltration, ssGSEA, WGCNA, LASSO

## Abstract

The current database has no information on the infiltration of glioma samples. Here, we assessed the glioma samples’ infiltration in The Cancer Gene Atlas (TCGA) through the single-sample Gene Set Enrichment Analysis (ssGSEA) with migration and invasion gene sets. The Weighted Gene Co-expression Network Analysis (WGCNA) and the differentially expressed genes (DEGs) were used to identify the genes most associated with infiltration. Gene Ontology (GO) and the Kyoto Encyclopedia of Genes and Genomes (KEGG) were used to analyze the major biological processes and pathways. Protein–protein interaction (PPI) network analysis and the least absolute shrinkage and selection operator (LASSO) were used to screen the key genes. Furthermore, the nomograms and receiver operating characteristic (ROC) curve were used to evaluate the prognostic and predictive accuracy of this clinical model in patients in TCGA and the Chinese Glioma Genome Atlas (CGGA). The results showed that turquoise was selected as the hub module, and with the intersection of DEGs, we screened 104 common genes. Through LASSO regression, TIMP1, EMP3, IGFBP2, and the other nine genes were screened mostly in correlation with infiltration and prognosis. EMP3 was selected to be verified *in vitro*. These findings could help researchers better understand the infiltration of gliomas and provide novel therapeutic targets for the treatment of gliomas.

## 1 Introduction

Glioma is a primary malignant tumor generated from glial cells in the central nervous system with the following characteristics: a high pathogenicity rate, a high disability rate, and a high recurrence rate (1). In clinical work, gliomas are usually classified into two types: (i) those with obvious borders and little penetration into adjacent brain tissue, such as pilocytic astrocytoma, subependymal giant cell astrocytoma, and pleomorphic xanthoastrocytoma, which have a favorable prognosis; (ii) another type of glioma is distinguished by the widespread infiltration and development of tumor cells into the surrounding brain tissue, without any discernible cytological or even imaging borders; the prognosis of these patients is usually poor (2, 3). The poor prognosis of glioma patients is partly due to the severely infiltrative features of glioma cells, which makes total surgical removal of gliomas impossible (4) and provides the prerequisites for glioma recurrence. Generally speaking, the diffuse infiltrative tumor cell is a key difficulty in the clinical management of glioma patients. The reality is that, in clinical work, we can assess the proliferation of gliomas by KI67 or PCNA, but we cannot make a better quantitative assessment of glioma infiltration. Accordingly, assessing the infiltration of samples may help doctors better treat and predict the prognosis of this disease (5).

On the other hand, glioma has significant heterogeneity, which may have different degrees of infiltration even at the same pathological level, particularly GBM (6); hence, assessing infiltration from pathology is difficult. As is known to all, magnetic resonance imaging (MRI) is the most common way to display the extent of glioma infiltration preoperatively (7); meanwhile, current databases lack this information in glioma samples. However, with the development of sequencing technology and transcriptome analysis algorithm, scientists can assess the infiltration status of immune cells in a sample, as well as the activation status of signaling pathways, through the transcriptome with Gene Set Enrichment Analysis (GSEA) (8). Scientists have constantly summarized different gene sets for different functions of cells, which include angiogenesis, proliferation, cell cycle, inflammation, migration, and invasion (9). Consequently, in the same way, we can assess the degree of infiltration of specimens through single-sample GSEA (ssGSEA) (10) based on the gene sets.

To find the marker genes related to the infiltration and survival of glioma, Weighted Gene Co-expression Network Analysis (WGCNA) was used to find the co-expressed genes linked to infiltration and prognosis (11); least absolute shrinkage and selection operator (LASSO) regression, which is the sum of the absolute values of all variable weights, was used to minimize features (12). Generally, the tens to hundreds of features are reduced to several or a dozen, and then the infiltration risk score is calculated for each patient according to the regression coefficient of the LASSO regression equation. We discussed the screened genes and verified the most likely gene *in vitro*.

However, many studies have been done on infiltration-related genes in glioma. In previous studies on individual genes, functional experiments can identify that it has more or less an effect on the infiltration of glioma (13, 14). It is just like looking for a needle in a haystack and not being able to assess its significance, and we rarely evaluate the function of genes on the whole. In this study, from the perspective of overall gene level and clinical model, combined with bioinformatics and experimental methodologies, we try our best to screen glioma infiltration-related genes. Finally, there are 12 genes found. We discover that EMP3 is an aberrant expression in the glioma cell line and determine how it contributes to the glioma migration and invasion. This research adds to our understanding of glioma infiltration and identifies novel therapeutic targets for glioma treatment and prognosis.

## 2 Materials and methods

### 2.1 Data acquisition and processing

The TCGA database (https://portal.gdc.cancer.gov, *n* = 702, accessed on 15 May 2022) provided the LGG and GBM RNAseq and clinical data. The CGGA database (http://www.cgga.org.cn, downloaded on 15 May 2022) has two datasets: mRNAseq 693 (*n* = 693) and mRNAseq 325 (*n* = 693), which contain RNAseq and clinical information on glioma in China. The gene set of migration (166 genes) and invasion (97 genes) was obtained from the Cancer Single-cell State Atlas (http://biocc.hrbmu.edu.cn/CancerSEA/goDownload).

### 2.2 Identifying infiltration-associated key genes

The ssGSEA algorithm quantifies the infiltration score of migration and invasion in the expression matrix (15). The WGCNA algorithm was used to screen the hub genes (11). The differential genes were calculated between high and low infiltration score samples. The common genes of hub genes and differential genes were chosen as candidate genes. The LASSO with the minimum cross-validation error is chosen to screen the key genes. The nomogram model was created to predict outcomes in 12-, 18-, and 24-month follow-ups in TCGA. The CGGA is an external dataset for validation.

### 2.3 GO, KEGG, and protein–protein interaction network analysis in the common genes

The cluster profile (R package) was used to perform GO enrichment and KEGG pathway analysis in common genes. The string_interactions of the common genes were downloaded from the STRING website and uploaded to Cytoscape. Proteins with important roles and their possible interactions are shown.

### 2.4 Mutation, methylation, and expression in glioma cell lines, and clinical relevance analysis of key genes

The maf data of GBM and LGG were downloaded from TCGA, and the maftools package is used to analyze the mutation of the key gene in R. The methylation of the genes was analyzed in CGGA. The RNAseq data of normal and glioma tumor cell lines were downloaded from the CCLE database to analyze the expression of key genes. The limma and SVA packages were used to remove batch effects. We calculated the survival curve of key genes and the correlation between key genes and the infiltration score.

### 2.5 Cell culture

The normal human astroglia (NHA), which were purchased from the Chongqing Golden Magpie Technology Development Co., and U251, LN229, A172, U118, and U87 cells, which were purchased from the China Center for Type Culture Collection (Shanghai, China), were cultured in the DMEM medium supplemented with 10% FBS (HyClone, UT, USA) and 1% penicillin–streptomycin (Beyotime Biotechnology, Jiangsu, China). The culture of cells followed a standard procedure. The media were replaced every 2 days.

### 2.6 Real-time PCR

RNAiso reagent (TaKaRa Biotechnology, Shiga, Japan) was used to extract total RNA from different cell lines according to the manufacturer’s recommendations. Complementary DNA was made by reverse transcription of total RNA (2 µg) using the HifairR III 1st Strand cDNA Synthesis SuperMix for qPCR kit (YEASEN) according to the manufacturer’s instructions. The HieffR qPCR SYBRR Green Master Mix (YEASEN) was utilized as a fluorescent dye, and the LightCyclerR 96 Real-Time PCR system (Roche) was employed. The following are the primers used: GAPDH: 5’-AATCCCATCACCTTCC-3’ (sense) and 5’-GAGTCCTTCCACGATACCAA-3’ (antisense); EMP3: 5’-GTCTCAGCCCTTCACATC-3’ (sense) and 5’-CAGCCAGCCATTCTCG-3’ (antisense); MMP-2: 5’-GACGGTAAGGACGGACTC-3’ (sense) and 5’-TGGAAGCGGAATGGAA-3’ (antisense); MMP-9: 5’-CCTGGAGACCTGAGAACC-3’ (sense) and 5’-GCAAGTCTTCCGAGTAGTTT-3’ (antisense). After an initial denaturation step of 95°C for 5 min, the tests were carried out in three different ways.

### 2.7 Western blotting analysis

Western blotting according to standard procedure. The difference is that the Trans-Blot SD Semi-Dry Electrophoretic Transfer Cell (Trans-BlotR TurboTM Transfer SYSTEM, BIO-RAD, USA) was then used to transfer total protein onto polyvinylidene fluoride membranes (Roche, USA). The following primary antibodies were used: anti-EMP3 (Abcam), α-tubulin, anti-MMP2, anti-MMP9, and anti-N-cadherin from Proteintech Group, Inc. ImageJ was used to perform densitometric analyses of immunoblots.

### 2.8 Small interfering RNA and cell

Transfection-specific siRNAs targeting EMP3 (siEMP3) and a negative control siRNA (siNC) were synthesized by TSINGKE (Beijing, China). The EMP3 siRNA sense and antisense sequences were as follows: siEMP3-1 (H2014-*siEMP3-1-ss: GGUACGACUGCACGUGGAATT, H2014-*siEMP3-1-as: UUCCACGUGCAGUCGUACCTT); siEMP3-2 (H2014-*siEMP3-2-ss: GCAGUAAUGUCAGCGAGAATT, H2014-*siEMP3-2-as: UUCUCGCUGACAUUACUGCTT); siEMP3-3 (H2014-*siEMP3-3-ss: CCUUCACAU-CCUCAUUCUUAU, H2014-*siEMP3-3-as: AUAAGAAUGAGGAUGUGAAGG). The siRNAs were transfected using the Liposomal Transfection Reagent (Hieff TransTM, YEASEN) according to the manufacturer’s instructions.

### 2.9 Cell proliferation, migration, and invasion assays

The growth curve assays of A172 and U118 cells, which were plated in 96-well plates at a density of 2 × 10^3^ per 200 μl, are according to standard procedure. In the colony formation assay, 1,000 cells from each group were plated into 12-well plates, where they were then cultivated for 10 days. The colonies with a diameter of more than 50 mm were counted. The 24-well Transwell chamber (Corning Incorporated, costarR, USA) with a membrane pore size of 8 µm was used for cell migration and invasion assays *in vitro*. The migration and invasion assays of A172 and U118 cells are according to standard procedure.

### 2.10 Statistical analysis

Bioinformatics-related data analyses were performed with R software version 4.0.1 (https://www.r-project.org). The results of assays are reported as means ± SD. All experiments were performed independently at least three times. Student’s *t*-tests or ANOVA was used to assess differences between groups, and *p* < 0.05 was considered statistically significant. For multiple analyses, the adjusted *p*-value was used. GraphPad Prism 5 (GraphPad Software, Inc., San Diego, CA, USA) was used for data analysis.

## 3 Results

### 3.1 Identification of the hub genes by WGCNA

**Figure 1:**
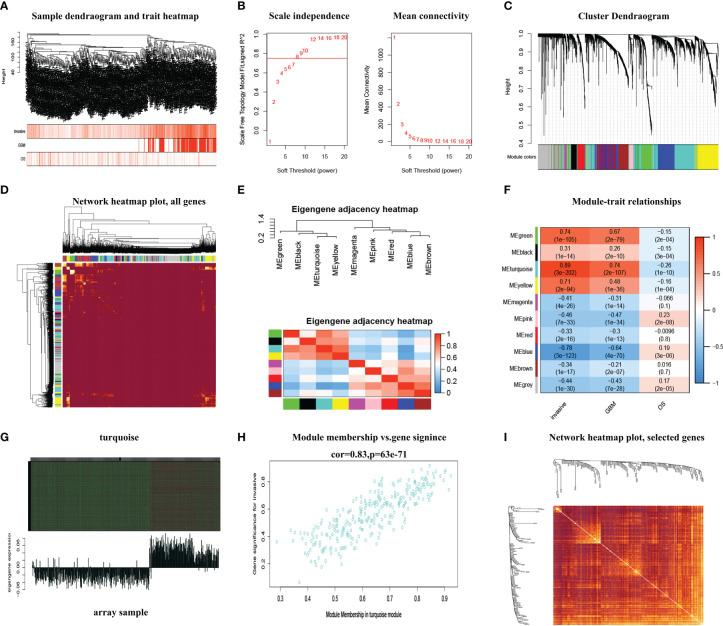
The WGCNA method was used to screen the hub genes. **(A)** Mapping of clinical trait variables and aggregation trees. **(B)** The soft thresholding and correlation coefficient. **(C)** The dynamic tree was constructed. **(D)** A TOM plot of genes in each module. **(E)** The eigengene adjacency heatmap. **(F)** Correlation between module eigengenes and GBM and overall survival rate. **(G)** Gene expression of the turquoise module with the feature vector of that module. **(H)** The scatter plots of module membership (MM) in the turquoise module versus gene significance (GS) of infiltration. **(I)** The TOM plot displays the topological relationship between different genes in the turquoise module.

Mapping of clinical trait variables and aggregation trees shows that the high infiltration score is not completely consistent with GBM and the overall survival rate (**Figure 1A**), which means that the WGCNA analysis based on the infiltration score and the overall survival rate is necessary. We selected *β* = 8 as the soft thresholding power to ensure a correlation coefficient close to 7.5 (**Figure 1B**). A total of 10 different color-coded co-expression modules were identified (**Figure 1C**). A topological overlap matrix (TOM) plot showed the topological relationship of genes in each module indicating that gene expression in each module was also relatively independent (**Figure 1D**). The eigengene adjacency heatmap shows a correlation between different modules; each module is relatively independent (**Figure 1E**). The turquoise module (which contained 1,180 genes) was selected as a significant module for further analyses (**Figure 1F**). Moreover, we generated scatter plots of module membership (MM) versus gene significance (GS) to show the relationship between them (**Figure 1G**), and a correlation was obtained between the turquoise module and infiltration (**Figure 1H**). Furthermore, in the turquoise module, the topological relationship between different genes is further demonstrated (**Figure 1I**). The top 25% of genes with the highest intro module connectivity, GS, and MM were chosen as hub genes.

### 3.2 The differentially expressed gene analysis between the high and low invasive glioma samples

**Figure 2:**
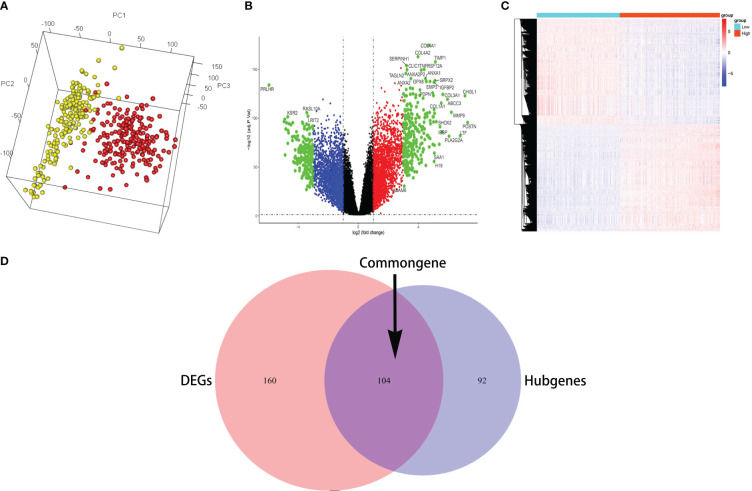
The common genes were identified. **(A)** The 3D PCA revealed the most homogeneity across tumor samples. **(B)** The volcano plot displayed the entire differential changes. **(C)** The clustering heatmap depicted the distribution of differentially expressed genes. **(D)** The Venn diagram displays the common genes between differential genes and hub genes.

The 3D PCA showed the highest homogeneity across tumor samples in high and low infiltration of glioma samples (**Figure 2A**). The volcano plot (**Figure 2B**) displayed the entire differential changes, with black dots representing genes with abs(logFC) < 1, *p* < 0.05; red and blue dots representing genes with 1 < abs(logFC) < 3, *p* < 0.05; and green dots representing genes with 3 < abs(logFC), *p* < 0.05. A total of 277 differentially expressed genes were filtered by logFC > 3 and *p* < 0.01, and the clustering heatmap depicted their distribution (**Figure 2C**). The common genes (which included 104 genes) between differential genes and hub genes are shown by the Venn diagram (**Figure 2D**).

### 3.3 GO, KEGG, and protein–protein interaction network analysis for the common genes

**Figure 3:**
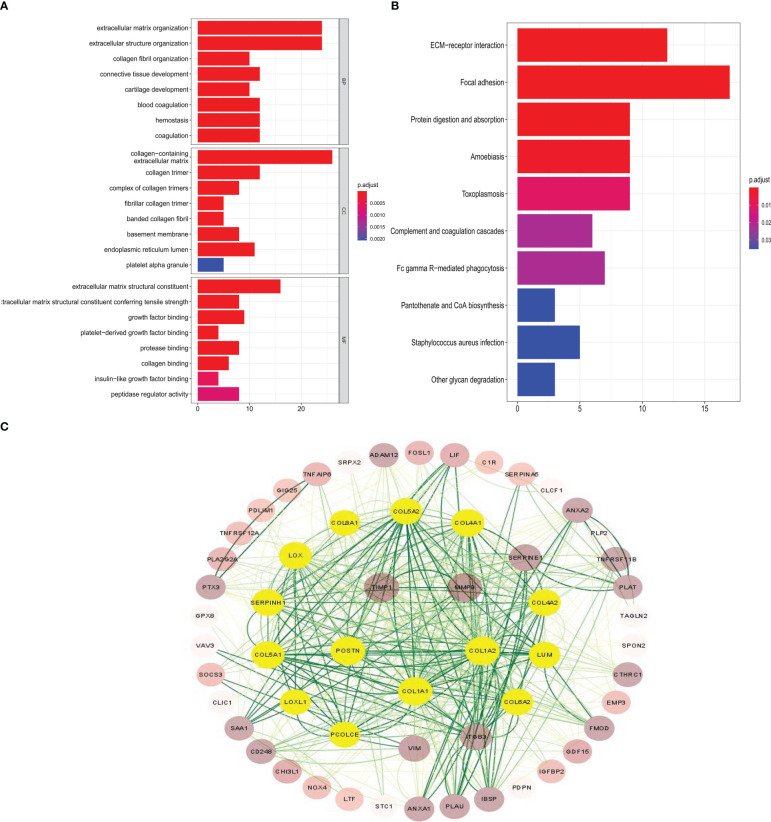
GO, KEGG, and protein–protein interaction network analysis in the common genes. **(A, B)** GO enrichment analysis and KEGG pathway analysis show the common genes participating in infiltration. **(C)** The most significant genes are based on the protein–protein interaction network in the common genes.

The common genes were selected for GO analysis; the BP, MF, and CC were mainly associated with the activation of extracellular matrix structural constituent, suggesting that the common gene is related to glioma infiltration to some extent (**Figure 3A**). To explore potential signaling pathways, KEGG pathways were employed to identify the function of the signaling pathway, ECM–receptor interaction, and focal adhesion may be the main signal pathway (**Figure 3B**). The protein–protein interactions (PPIs) of important genes were depicted in the PPI network, and a module consisting of 14 genes was recognized as a significant cluster in the PPI network (**Figure 3C**). The most significant genes are based on the PPI network in the common genes. The shade of the green line represents the possibility of interaction between molecules; the darker the color, the higher the possibility. The shades of brown in the circle represent the number and heighten the ability of interactions between different molecules, whereas light yellow indicates the molecules most likely to interact with each other.

### 3.4 LASSO regression analysis identification of key genes and nomogram construction and validation

**Figure 4:**
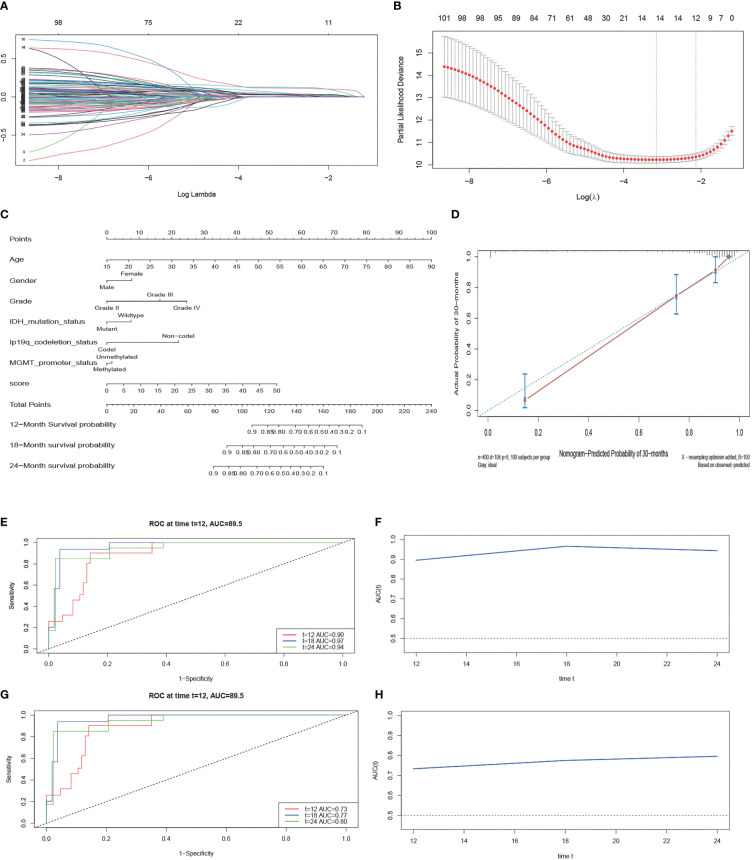
LASSO regression analysis and construction and calibration of the nomogram. **(A, B)** Twelve candidate genes screened out by LASSO with the minimum cross-validation error. **(C)** Nomogram integrating risk score and clinical characteristics. **(D)** Calibration of the nomogram at 30-month survival in the training cohort. **(E, F)** The AUC of time-dependent ROC at 12-month, 18-month, and 24-month survival in the test cohort. **(G, H)** The AUC of time-dependent ROC at 12-month, 18-month, and 24-month survival in the external cohort.

When the *λ* value is 0.000176, the model has 101 genes (**Figure 4A**). Following that, we use K-fold cross-validation that yielded a model with a *λ* value of approximately 0.1183128; the 101 genes were shrunk to 12 genes (**Figure 4B**). These gene features were presented as an invasive risk score in the calculation formula. Invasive risk score = (0.024716727 × expression level of TIMP1) + (0.001692998 × expression level of EMP3) + (0.104612161 × expression level of IGFBP2) + (0.003649724 × expression level of CHI3L1) + (0.028455608 × expression level of TUBA1C) + (0.109404724 × expression level of RAB42) + (0.027050097 × expression level of SH2D4A) + (0.018645393 × expression level of SHOX2) + (0.001780754 × expression level of PTX3)+ (0.011420430 × expression level of HOXA1) + (0.032166703 × expression level of HOXA3) + (0.022096648 × expression level of HOXD11). To verify the reliability of the screened genes, we also calculated patients’ risk score in the CGGA cohort with the same formula. Next, we randomly divided the dataset of TCGA into a training set and a test set after multivariate analysis of combined risk score and common clinical features. The result shows that risk score is an independent risk factor (**Table 1**). The nomogram integrated both the risk score and clinicopathological risk factors constructed in the training set (**Figure 4C**). The C-index is 0.88 and the calibration curve showed that the nomograms did well compared with an ideal model (**Figure 4D**). Second, we assessed the prognostic accuracy of the Cox regression model with time-dependent ROC analysis at varying follow-up times (**Figures 4E, F**) in the test set. Twelve-month survival was 0.90, 18-month survival was 0.97, and 24-month survival was 0.94. We did the same analyses using tissue samples from the external dataset cohort—the CGGA dataset; 12-month survival was 0.73, 18-month survival was 0.77, and 24-month survival was 0.80 (**Figures 4G, H**). After LASSO screening and clinical model validation, the 12 genes were selected as the most related to glioma invasion and clinical prognosis.

**Table 1:** Multifactorial analysis of risk-score and common clinically features.

Variables	CGGA(Multivariate analysis)		Variables	TCGA (Multivariate analysis)	
	HR(95% Cl)	p value		HR(95% Cl)	p value
Age(years)			Age(years)		
>50(N=268)/<50(N=701)	1.12(0.91-1.4)	p=0.27	>50(N=258)/<50(N=354)	2.96(1.85-4.8)	p<0.001
Gender			Gender		
Male(571)/Female(N=399)	0.97(0.81-1.2)	p=0.747	Male(349)/Female(N=254)	0.96(0.68-1.4)	p=0.814
Grade			Grade		
Grade II(N=270)	2.66(1.98-3.6)	p<0.001	Grade II(N=213)	0.95(1.14-3.3)	p=0.015
Grade III(N=322)	2.66(1.98-3.6)	p<0.001	Grade III(N=238)	1.95(1.14-3.3)	p=0.015
Grade IV(N=374)	4.47(3.24-6.2)	p<0.001	Grade IV(N=152)	1.50(1.20-5.2)	p=0.015
IDH_mutation_status			IDH_mutation_status		
Wildtype(N=421)/ Mutant(N=500)	0.99(0.76-1.3)	p=0.937	Wildtype(N=224)/ Mutant(N=373)	1.65(0.82-3.3)	p=0.159
Ip/ 19q_codeletion_status			Ip/ 19q_codeletion_status		
Non-codel(N=697)/ Codel(N=199)	2.70(1.96-3.7)	p<0.001	Non-codel(N=449)/ Codel(N=149)	1.88(1.00-3.5)	p=0.051
MGMTp_methylation_status			MGMTp_methylation_status		
methylated(N=456)/ un-methylated(N=361)	1.12(0.93-1.4)	p<0.221	methylated(N=148)/ un-methylated(N=425)	1.04(0.70-1.5)	p=0.845
Risk-score(N=970)	1.04(1.02-1.1)	p<0.001	Risk-score(N=603)	1.05(1.02-1.1)	p<0.001

HR, Hazard Ratio; CI, Confidence Interval.

### 3.5 The expression of the key genes is closely related to clinical features

**Figure 5:**
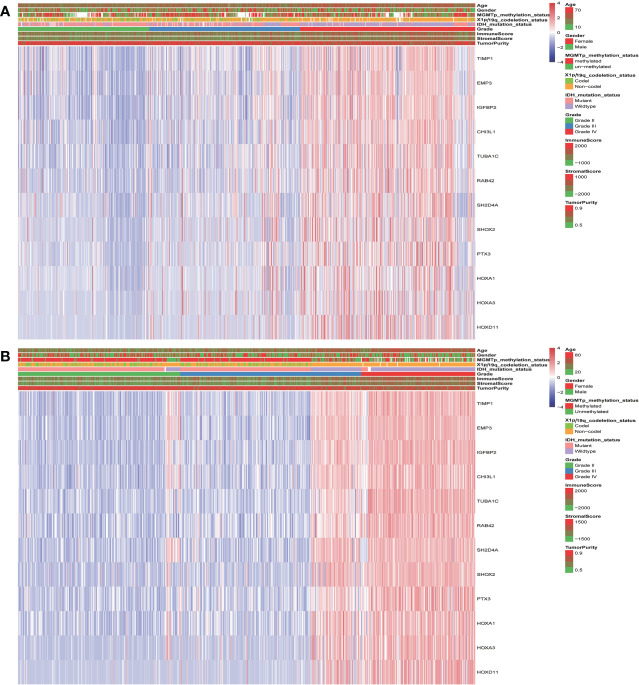
The landscape of glioma clinical, immune score, and molecular characteristics in association with the expression of key genes in TCGA **(A)** and CGGA **(B)**.

The landscape of clinical and molecular characteristics is described in TCGA and CGGA, respectively (**Figures 5A, B**). The expression of the key genes increased with the increase of tumor grade and was closely related to IDH mutation and 1p/19q codeletion. Although it has little correlation with stromal, immune scores and tumor purity, the MGMT promoter mutation did not exhibit this trend. Furthermore, the expression of key genes shows no significant difference in terms of gender and age.

### 3.6 Genomic alterations and methylation analysis of the key genes in glioma samples

**Figure 6:**
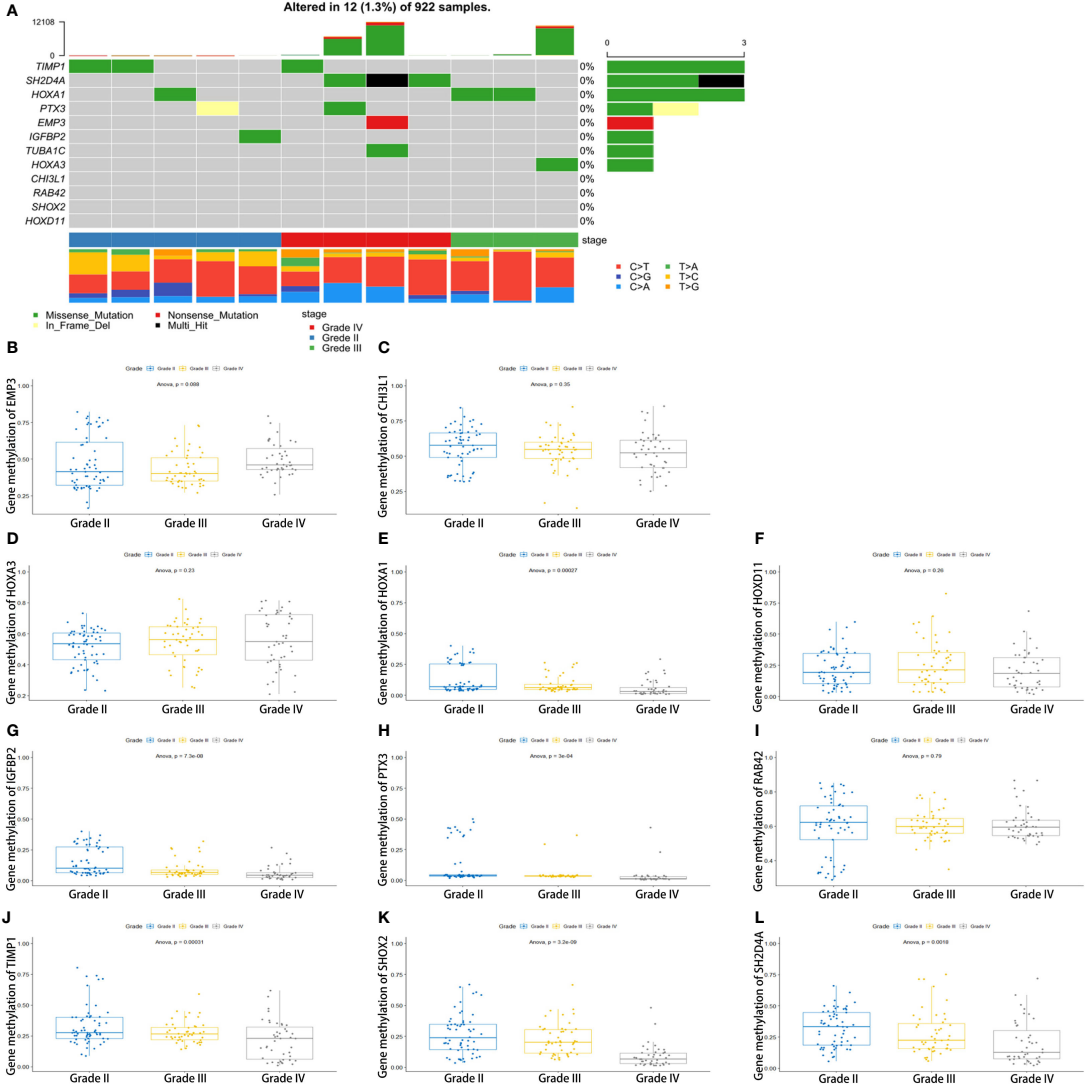
Genomic alterations and methylation analysis of the key genes in glioma samples. **(A)** The somatic mutation of the 12 genes. **(B–L)** The methylation analysis of the 12 genes.

To determine the reason for the alteration of these genes, we performed somatic mutation analyses using the TCGA database. We found that the somatic mutation of 12 key genes is very low (**Figure 6A**). The expression alteration of these genes may be attributable to other reasons. These genes are not glioma promoters. The methylation of HOXA1, IGFBP2, PTX3, TIMP1, SHOX2, and SH2D4A decreases with increasing tumor grade. There was also no significant difference in the methylation of other genes (**Figures 6B–L**).

### 3.7 The key genes were significantly correlated with the infiltration and prognosis of glioma

**Figure 7:**
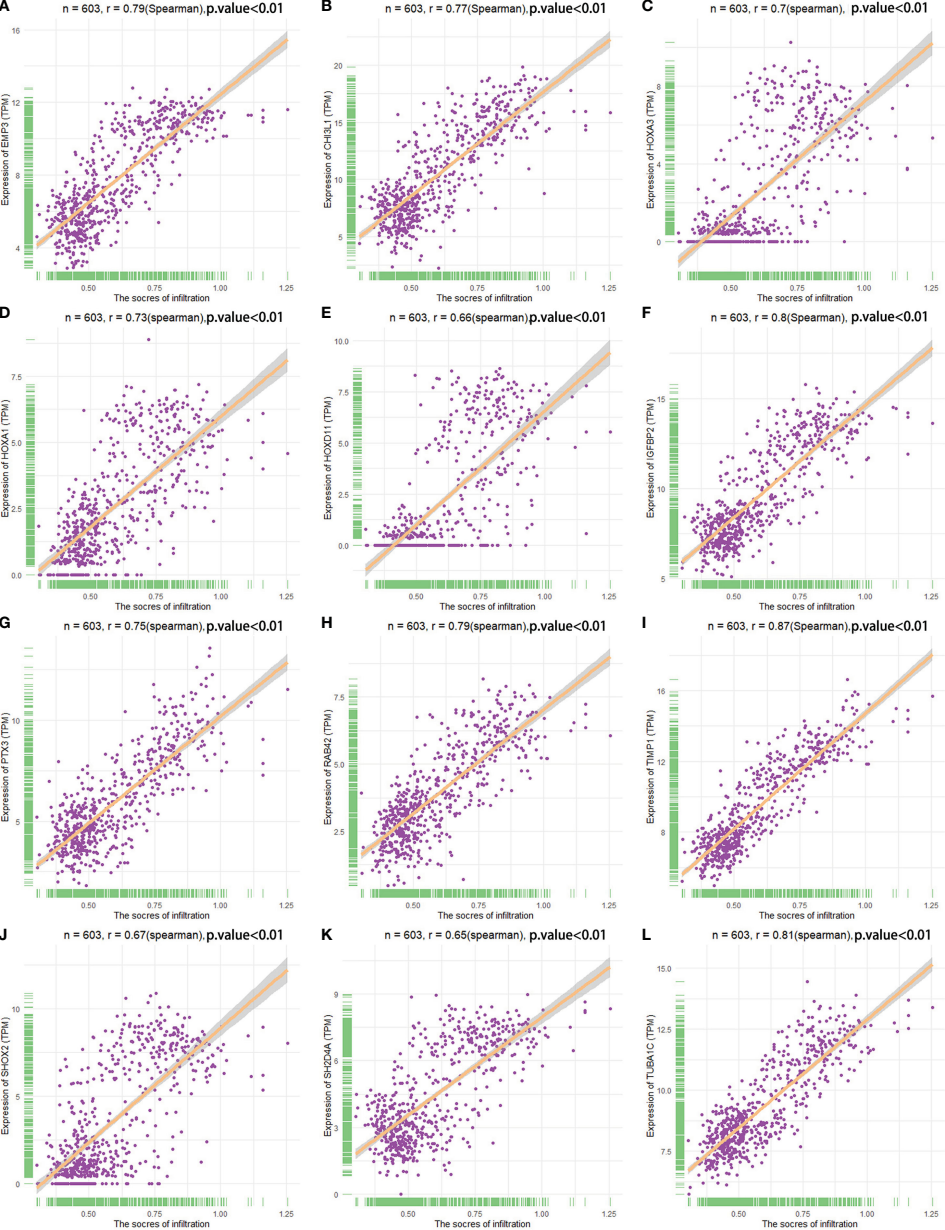
Spearman correlation analysis analyzed the correlation between key genes and infiltration score. **(A–L)** The relationship between the key genes and infiltration scores.

**Figure 8:**
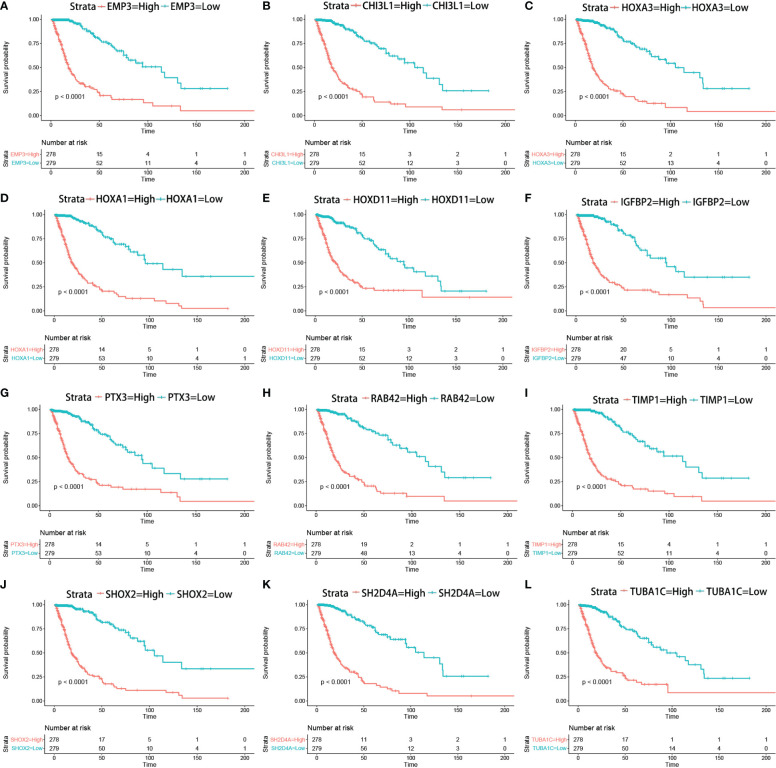
Kaplan–Meier survival curves in glioma patients in TCGA show that the expression levels of key genes predicted prognosis **(A–L)** (Kaplan–Meier survival analysis, *p* < 0.0001 in log rank).

The correlation between the expression of the key genes and infiltration was calculated (**Figures 7A–L**). All the key genes were highly associated with infiltration. Moreover, analysis of TCGA patient survival after segregation revealed that elevated key gene expression predicted prognosis (**Figures 8A–L**). These data suggest that the key genes play a crucial role in the malignant process of glioma.

### 3.8 EMP3 is significantly expressed in glioma cell lines

**Figure 9:**
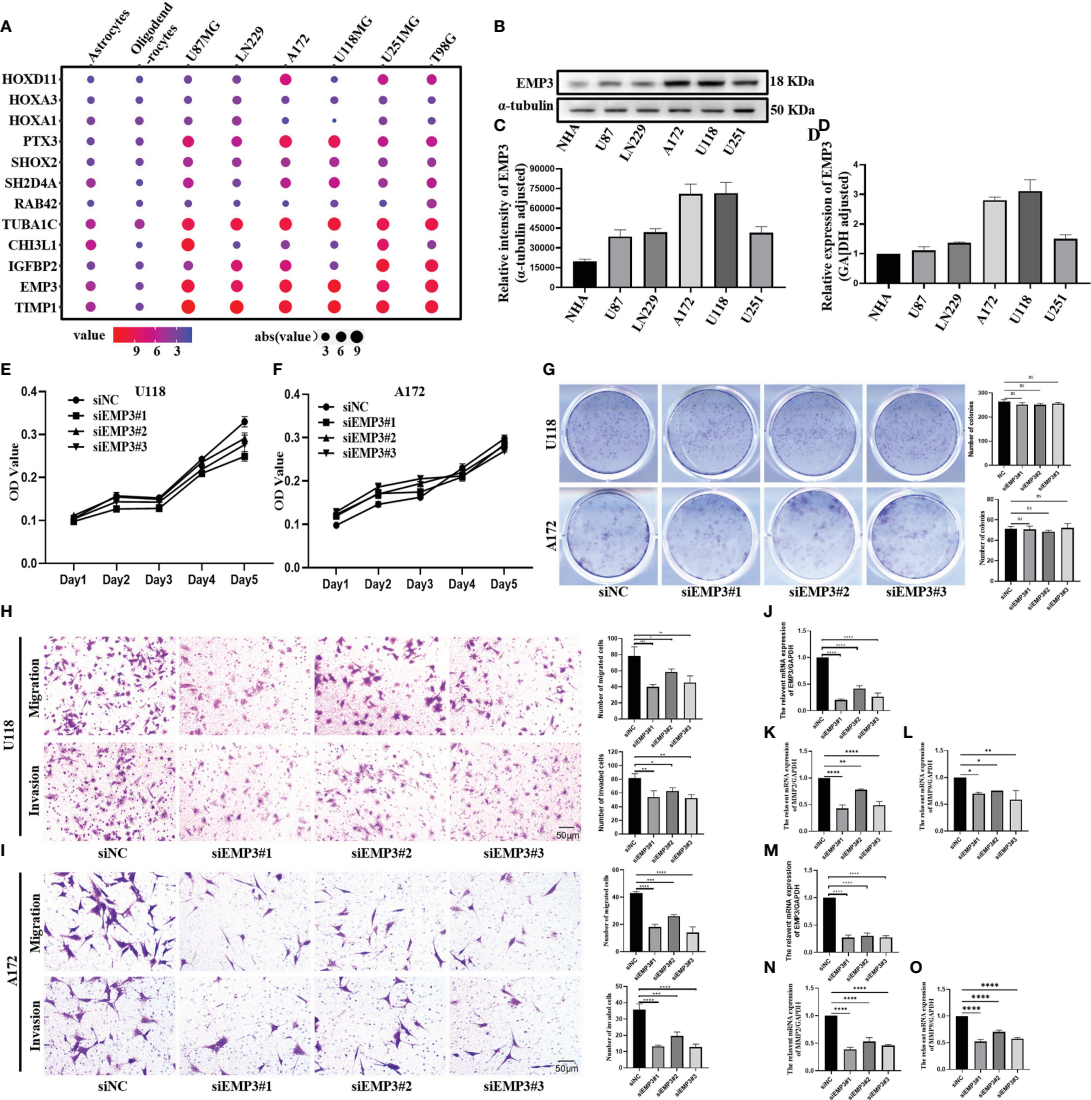
EMP3 silencing attenuates the migration and invasion of glioma cells. **(A)** The expression of EMP3 in different cell lines in the CCLE datasets. **(B–D)** Western blot and qPCR display EMP3 express highly in U118 and A172. **(E–G)** The growth curve and plate cloning experiment display that EMP3 silencing has little effect on the cell proliferation of U118 and A172. **(H, I)** EMP3 knockdown inhibits the migration and invasion of glioma cells. (**J–O**, 20×) qPCR displays EMP3 silencing attenuates the expression of MMP2 and MMP9 expression (ns, *p* > 0.05; *p < 0.05; **p < 0.01; ***p < 0.001; ****p < 0.0001).

**Figure 9A** shows that PTX3, TUBA1C, EMP3, and TIMP1 were highly expressed in glioma cell lines. IGFBP2 is highly expressed in U251MG, A172, and T98; conversely, it is lowly expressed in U118 and U87. CHI3L1 is only highly expressed in U87 and U118, and HOXD11 is only highly expressed in U251, A172, and T98G. Moreover, SHOX2 and SH2D4A were highly expressed in glioma cell lines, but the difference was not obvious. There was no significant difference in the expression of HOXA3, HOXA1, and RAB42 between glioma cell lines and normal cells. Combined with the result of the PPI network, We select EMP3 for the next verification step.

### 3.9 siEMP3 knocks down the expression of EMP3 in A172 and U118

**Figure 10:**
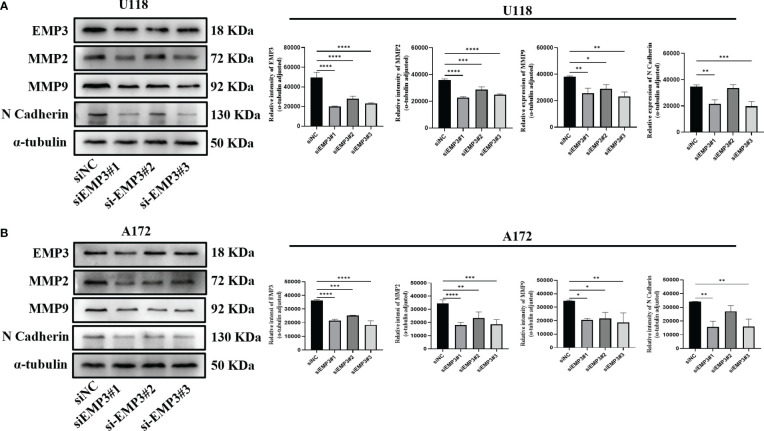
EMP3 silencing suppressed EMT marker expression. **(A)** Western blot analysis of U118 transfected with indicated siRNAs targeting EMP3 (siEMP3#1, siEMP3#2, and siEMP3#3) or siNC. **(B)** Western blot analysis of A172 transfected with indicated siRNAs targeting EMP3 (siEMP3#1, siEMP3#2, and siEMP3#3) or siNC. (p > 0.05; *p < 0.05; **p < 0.01; ***p < 0.001; ****p < 0.0001).

In comparison to NHA, EMP3 was considerably expressed at the mRNA and protein levels in U118 and A172 (**Figures 9B–D**); therefore, we selected U118 and A172 cells to study the role of EMP3. Compared with siNC (negative control), the EMP3 mRNA levels in the siRNA group decreased by 79.6%, 58.1%, and 73.6% in U118 cells and by 72.9%, 69.7%, and 72.2% in A172 (**Figures 9J, M**, ****p < 0.0001); Western blots revealed that EMP3 protein levels decreased by 73.8%, 54.5%, and 66.6% in U188 and by 33.1%, 19.3%, and 36.9% in A172 (**Figures 10A, B**, ***p < 0.001; ****p < 0.0001).

### 3.10 EMP3 is required for glioma cell migration and invasion

The MTT assay revealed that the growth curve in the siEMP3 group did not show a time-dependent delay in growth when compared to the negative group cells (**Figures 9E, F**); the colony formation assay also revealed that EMP3 silencing had no significant effect on A172 and U118 growth on day 10 (**Figure 9G**). In the cell migration experiment, EMP3 silencing inhibited cell migration compared to the negative group in both U118 and A172 cells (40.07 ± 3.06 vs. 78.73 ± 11.05, 58.40 ± 3.80 vs. 78.73 ± 11.05, and 45.40 ± 5.24 vs. 78.73 ± 11.05 in U118 and 18.07 ± 2.02 vs. 43.20 ± 1.06, 26.00 ± 1.25 vs. 43.20 ± 1.06, and 14.07 ± 4.23 vs. 43.20 ± 1.06 in A172, respectively) (**Figures 9H, I**, *p < 0.05;**p < 0.01;***p < 0.001;****p < 0.0001). The invasion of U118 and A172 also decreased in siEMP3 groups compared to the negative group (65.87 ± 3.25 vs. 81.47 ± 6.80, 62.87 ± 4.91 vs. 81.47 ± 6.80, and 52.40 ± 5.29 vs. 81.47 ± 6.80 in U118 and 13.27 ± 0.81 vs. 19.53 ± 2.50, 19.53 ± 2.50 vs. 35.80 ± 3.53, and 12.73 ± 1.81 vs. 35.80 ± 3.53 in A172, respectively) (**Figures 9H, I**, *p < 0.05;**p < 0.01;***p < 0.001;****p < 0.0001). Consistent with the above phenomenon, qRT-PCR revealed that MMP-2 and MMP-9 mRNA levels in the siEMP3 groups decreased by 57.3%, 22.15%, and 51.05%, and 30.27%, 25.01%, and 41.61% in U118 and by 61.24%, 46.81%, and 54.07%, and 47.71%, 29.82%, and 42.54% in A172, respectively, compared to the negative group (**Figures 9K, L, N, O**, *p < 0.05; **p < 0.01;****p < 0.0001). Western blots also revealed that the protein levels of MMP-2 and MMP-9 in siEMP3 groups decreased by 36.26%, 19.68%, and 30.32%, and 31.88%, 23.20%, and 38.81% in U118 and by 47.48%, 32.51%, and 45.96%, and 40.07%, 37.36%, and 46.12% in A172 (**Figures 10A, B**, *p < 0.05;**p < 0.01;***p < 0.001;****p < 0.0001). We also determined the glioma cells transfected with siRNAs targeting EMP3, which showed that the expression of several EMT markers including N-cadherin was downregulated by EMP3 depletion (**Figures 10A, B**, **p < 0.01; ***p < 0.001) in U118 and A172. In summary, the knockdown of EMP3 expression inhibited the migration and invasion of glioma cells.

## 4 Discussion

In clinical work, the most important thing that comes to mind for neurosurgeons is how to resect the glioma to the maximum degree possible. However, the glioma boundary, like the root silk of tree roots, is not apparent, indicating infiltrative growth (16). It is difficult to complete removal of the tumor and the residual glioma cells relapsed sooner (17). In recent years, neurosurgeons have used a variety of auxiliary technologies to improve the accurate judgment of tumor boundaries during surgery, such as intraoperative MR, intraoperative B-ultrasound, neuronavigation technology, and fluorescence guidance technology (18), whereas the essence of the infiltration features and mechanism of glioma at the molecular level still needs further in-depth study. Much research on the invasiveness of glioma is now underway; our goal is to use new algorithms and clinical modeling pathways (19) to identify the molecular markers most associated with glioma infiltration and prognosis and to help neurosurgeons assess the glioma infiltration in clinical work and provide a new target for the treatment of glioma.

Consistent with our research, the biological function of some of the key genes we screened has been investigated in previous studies. According to a growing number of studies, the tissue inhibitor of metalloproteinases 1 (TIMP1) has been related to a poor prognosis in a range of malignancies (20–23). Aaberg-Jessen et al. (24) discovered that TIMP1 expression has been connected to the grade and prognosis of glioma. In the patients with GBM, a high serum level was reported to be a poor prognostic predictor (25). In metastatic carcinoma and glioma cells, TIMP1 has been demonstrated to interact with P75NTR (26), TIMP-1, and CD63, and may play a role in glioblastoma stemness (27). TIMP-1, on the other hand, is one of the natural inhibitors of matrix metalloproteinases (MMPs) as a member of the TIMP gene family. The expression pattern of TIMP-1 and its molecular function in a tumor are conflicting. According to the current research, what can explain this phenomenon is that TIMP1 is not only an MMP inhibitor but also a multipotent protein that supports other processes of invasion and migration. It has other important functions in cancer as well: TIMP1 may stimulate cell growth (28), regulate angiogenesis (29), and inhibit apoptosis (30). Insulin-like growth factor-binding protein 2 (IGFBP2) is a member of the IGFBP family and functions by binding to IGF or in an IGF-independent way (31). Studies over the last decades have shown that IGFBP2 is upregulated and promotes several key oncogenic processes and is highly expressed in many malignancies (32). As a candidate biomarker, the aberrant expression of IGFBP2 was detected in high-grade gliomas and identified as a signature associated with poor prognosis (33). Several studies have validated IGFBP2’s possible roles in glioma initiation, proliferation, invasion, and chemoresistance to temozolomide (34, 35). Additionally, Cai et al. also found that IGFBP2 is also engaged in immunosuppressive activities and is an independent adverse prognostic biomarker for GBM patients, suggesting that IGFBP2 is critical in the glioma tumor microenvironment (36).

Chitinase-3 like-protein-1 (CHI3L1) is a member of the glycoside hydrolase family 18, which is synthesized and secreted by a variety of cells, including some inflammatory cells, connective tissue cells, and tumor cells (37). To date, CHI3L1 is overexpressed in a variety of human and animal tumor models, and it is currently being considered a potential diagnostic marker and therapeutic target in oncology (26, 37). Overexpression of CHI3L1 is associated with a poor prognosis in glioblastoma patients, and it is involved in cancer cell growth, proliferation, invasion, metastasis, and angiogenesis (38). Additionally, it was discovered by Chen et al. that CHI3L1 reprograms tumor-associated macrophages, which alters an immune-suppressive milieu (TAMs) (39). Tubulin alpha-1c chain (TUBA1C) is an isoform of α-tubulin. Current research suggests that the TUBA1C gene has the potential as a biomarker for tumor prognosis and immunotherapy outcomes (38). It is involved in the cell cycle, cell proliferation and migration, and the immune microenvironment. Furthermore, TUBA1C has been reported to be associated with glioma, and its expression promotes proliferation by regulating the cell cycle and denotes poor prognosis in glioma (40, 41). RAB42 is a member of the mammalian Rab family of small GTPases (42) and has been attributed to prognosis in glioma patients (43). Subsequently, following that, Liu et al. confirmed that RAB42 increased glioma proliferation, migration, and invasion and that RAB42’s pro-oncogenic mechanism is linked to the activation of VEGF signaling pathways (44). Src homology 2 domain-containing 4A (SH2D4A) is located on chromosome 8p, which is commonly deleted in various cancer entities including breast cancer, prostate cancer, and hepatocellular carcinoma (45–48) and has been shown to correlate with poor patient survival. Current studies show that SH2D4A may be a suppressor gene and prevents the nuclear translocation of the pro-tumorigenic transcription factor STAT3 (49). At present, there are few studies on glioma about SH2D4A. The SHOX2 gene, which is found on chromosome 3q and encodes a transcriptional regulator, is a member of the homeobox family of genes whose expression is confined to craniofacial, brain, heart, and limb development (50). For non-small cell lung cancer patients, SHOX2 promoter DNA methylation has been discovered as a diagnostic and prognostic biomarker (51). SHOX2 overexpression has been linked to hepatocellular carcinoma tumor recurrence (52) and poor breast cancer survival (53). SHOX2, as a single indicator or in combination with IDH and other biomarkers, is used to improve survival predictions for LGG patients (54).

The glycoprotein pentraxin-3 (PTX3) is a prototypic member of the long pentraxin subfamily that has an unrelated long N-terminal domain coupled to the C-terminal domain, and is used for gene organization, cellular source, tissue source, inducing stimuli, and ligand recognition (55). PTX3 is correlated with various malignancies. Current studies show that overexpression of PTX3 in glioblastoma cells is associated with increased invasion and the IL8–VEGF signaling axis (56). PTX3 is a component of the glioma microenvironment, being generated by tumor cells and infiltrating CD68-positive macrophages, and local PTX3 levels are correlated with grade and malignancy (57). In addition, Tung et al. and Liu et al. have identified that PTX3 plays a crucial role in glioma cell proliferation and invasion (58, 59), and may thus serve as a novel potential therapeutic target in the treatment of gliomas; it is related to the recurrence of glioma (60). HOXA1 is a DNA-binding transcription factor that regulates gene expression, morphogenesis, and differentiation. It is found on chromosome 7 as part of the A cluster homeobox genes. Current investigation shows that the long noncoding RNA HOTAIRM1 facilitated GBM proliferation and invasion by interacting with DNA methyltransferases (Dnmts) EZH2 and G9a and sequestering them away from the HOXA1 gene’s transcription start sites (TSS), therefore promoting the HOXA1 oncogene expression (61). HOXD11 is a member of the HOX gene family, which encodes transcription factors that regulate various physiological processes. HOXD11 has been associated with several malignancies in recent years, including laryngeal squamous cell carcinoma, ovarian cancers, and head and neck cancer (62–64). Furthermore, HOXD11 can promote the malignant behavior of glioma cells as an oncogene by participating in the regulation of cell cycle signaling pathways (64). However, its invasion-related function has not been studied.

There is clear evidence that PTX3, TIMP1, and TUBA1C are related to glioma invasion. However, the function of EMP3 remains unclear. The current consensus is that EMP3 is a tumor suppressor gene, whereas the current database shows that EMP3 is significantly expressed in GBM and is related to the prognosis of gliomas. Its specific function in glioma needs to be further investigated. EMP3 belongs to the PMP-22/EMP/MP20 protein family (65). The protein contains four transmembrane domains and two N-linked glycosylation sites. It is thought to play a role in cell proliferation and cell–cell interactions, and function as a tumor suppressor (66). Early studies on EMP3 showed that EMP3 CpG island hypermethylation was found to be an indication of poor prognosis in neuroblastoma patients. Overexpression of EMP3 significantly inhibited the growth of G418 *in vivo* and *in vitro* (67). Zhou et al. also discovered that EMP3 is a possible tumor suppressor in breast cancer, inhibiting S-phage entrance, DNA replication, DNA damage repair, and stem-like features, and EMP3 downregulation may be responsible for breast cancer chemoresistance (68). However, compared with normal brain tissue, EMP3 was significantly overexpressed in GBM, but low in LGG, and there was no significant difference between LGG and normal brain tissue in TCGA. The expressive property of EMP3 is opposite to its function in GBM. To investigate the function of EMP3 in GBM, we performed knockdown experiments with siRNAs and found that EMP3 knockdown inhibits the migration and invasion of glioma, but did not affect the proliferation of glioma. Furthermore, we discovered this behavior at the mRNA and protein levels, with EMP3 knockdown downregulating MMP2 and MMP9, following the same pattern as N-cadherin. There are three potential causes for this phenomenon: EMP3 is a tumor suppressor gene by itself, and its expression levels may increase along with the substrate that is being repressed; second, EMP3 is a tumor suppressor gene at low expression levels and an oncogene at high expression; third, EMP3 is an oncogene in glioma; however, there may be additional causes. Further research is needed to investigate this mechanism.

Interestingly, according to current research, the critical genes that we screened for invasive characteristics also have a role in the immunological microenvironment of glioma. Chen et al., for example, identified that EMP3 mediates glioblastoma-associated macrophage infiltration to drive T-cell exclusion (69); Chen et al. discovered that Chitinase-3-like 1 protein complexes modulate macrophage-mediated immune suppression in glioblastoma (39); and Locatelli et al. revealed that PTX3 may represent a new marker of cancer-related inflammation and glioma malignancy (57). This phenomenon, however, interests us, and we hypothesize that the rationale is that glioma invasion is also a part of the glioma microenvironment, and perhaps these key genes can also be used to visualize inflammation and tumor (70). Meanwhile, we came upon some perplexing phenomena. For instance, consider the action of TIMP1, an MMP inhibitor. It should theoretically limit glioma invasion, yet its expression is positively associated with glioma grade and adversely correlated with glioma prognosis. This might be because invasion is independent of glioma grade, or that TIMP1 as a multipotent protein promotes glioma invasion and migration through different mechanisms. Furthermore, a previous study has discovered that hypermethylation of EMP3’s promoter silences its production in glioma epigenetically and exhibits tumor suppressor features in glioblastoma (67). However, we observed that, in the CGGA database, there was no significant difference in methylation of the EMP3 gene among grades, and that EMP3 expression increased with glioma grade. Based on our observations and tests, we determined that EMP3 may still be an oncogene in glioma. Methylation of the EMP3 promoter is one way how its expression is controlled; however, it is not the most important determinant in EMP expression.

In this study, combined with bioinformatics and a clinical model, from the perspective of the invasion and migration of glioma, there are 12 genes found. According to the current research, some of the genes found have been verified. However, we looked into the role of EMP3 in glioma cells and confirmed our conclusion. In the future, we will be able to measure the level of key gene expression in glioma tissues by immunohistochemistry or sequencing to assess tumor infiltration and develop more effective clinical decision-making methods. We can also utilize important genes as targets to create medications to cure them, and of course, there is still a lot of research that needs to be done and more mechanisms that need to be thoroughly investigated. To summarize, this study adds to our understanding of glioma infiltration, and it may open up new avenues for the investigation of molecular features and targeted therapeutics for glioblastoma.

## Data availability statement

The original contributions presented in the study are included in the article/**Supplementary Material**. Further inquiries can be directed to the corresponding author.

## Author contributions

For research, SS designed the general idea, and all the data of the article were processed and analyzed by SS. SS and JZ were jointly responsible for the completion of the experiment, and WP, HY, and DZ put forward valuable suggestions for the writing of the paper. HC and XS were responsible for the quality control of the article and guided the entire analysis process. All authors contributed to the article and approved the submitted version.
